# Serum High-sensitive C-Reactive Protein, Interleukin-6 and Malondialdehyde Levels in Acne Vulgaris and Their Correlation With Disease Severity: A Cross-Sectional Study

**DOI:** 10.7759/cureus.88405

**Published:** 2025-07-21

**Authors:** Sumitra Bhoi, Kuldip Das, Purnima Meher, Bharati Panda, Madhusmita Acharya, DibyaPrakash Barik, Mamata Pandey

**Affiliations:** 1 Biochemistry, Veer Surendra Sai Institute of Medical Science and Research, Burla, IND; 2 Dermatology, Veer Surendra Sai Institute of Medical Science and Research, Burla, IND; 3 Physiology, Veer Surendra Sai Institute of Medical Science and Research, Burla, IND; 4 Community Medicine, Veer Surendra Sai Institute of Medical Science and Research, Burla, IND; 5 Multidisciplinary Research Unit, Veer Surendra Sai Institute of Medical Science and Research, Burla, IND

**Keywords:** acne vulgaris, chronic inflammation, food habits, gags score, gender comparison, hs-crp, il-6, life style habits, mda, pustular lesion

## Abstract

Background

Acne vulgaris (AV) is a chronic inflammatory skin condition primarily affecting adolescents and young adults. It is a multifactorial disorder characterized by the formation of open and closed comedones, papules, pustules, nodules, and cysts. Emerging evidence suggests that reactive oxygen species (ROS), especially lipid peroxides (LPO), play a role in mediating acne inflammation. Malondialdehyde (MDA) is one of the byproducts of lipid peroxidation commonly used as a biomarker of cell damage.

Objectives

The primary objective of this study was to estimate inflammatory markers such as high-sensitive C-reactive protein (Hs-CRP), interleukin-6 (IL-6) and an oxidative stress marker, malondialdehyde (MDA), in acne patients. The secondary objective was to find out their association with the Global Acne Grading System (GAGS) score.

Methodology

The cross-sectional study was conducted in the Department of Dermatology in collaboration with the Department of Biochemistry at the Veer Surendra Sai Institute of Medical Science and Research in Burla, Odisha. The study period was from August 2023 to January 2024. Participants aged 15-35 years of either sex having acne vulgaris (sample size 150) were examined clinically and severity of acne was evaluated by GAGS score.

Results

The present study included 69 men and 81 women. The mean age of men and women was 20.59±1.3 and 22.25±0.4 years, respectively. The mean Hs-CRP (2.40±0.52 mg/L), IL-6 (21±0.47 pg/ml) and MDA levels (3.57±1.31 nmol/ml) were higher in men than in women. The Hs-CRP (3.0±0.10 mg/L), IL-6 (8.75±1.63 pg/ml) and MDA (5.31±1.89 nmol/ml) levels were higher in the severe group than in moderate and mild groups with significance (p<0.05). Also, highly significant positive correlation of Hs-CRP (r= 0.137, p-value 0.009), IL-6 (r= 0.719, p-value 0.008) and MDA (r= 0.316, p- value 0.007) was observed when compared to mild and moderate groups.

Conclusion

There were differences in Hs-CRP, IL-6 and MDA levels based on the severity of acne vulgaris.

## Introduction

Acne vulgaris (AV) is a common dermatological condition that manifests as various non-inflammatory and inflammatory lesions. It is predominantly found on the face, chest, back, and upper arms [1]. Acne vulgaris primarily affects adolescents and young adults, with a global prevalence ranging from 35% to 90% [2]. Severe acne often results in scarring that can significantly impact the quality of life and psychological well-being [3,4]. Although the exact etiology remains unclear, contributing factors may include follicular hyperkeratinization, local release of pro-inflammatory chemical mediators, increased sebum production, hormonal influences, and colonization by *Cutibacterium acne* [5]. Emerging evidence suggests that oxidative stress plays a dominant role in the pathogenesis of acne, which results from an imbalance between free radical generation and the body’s antioxidant defence mechanism [6]. It has been hypothesized that colonization of *C. acne* can cause the accumulation of neutrophils, releasing proteases, lipases, and reactive oxygen species (ROS), leading to lipid peroxidation and subsequent inflammation [7,8]. Proinflammatory cytokines such as interleukin-1 (IL-1), IL-6, and tumor necrosis factor (TNF)-α, are known modulators of inflammatory responses in acne [9]. IL-6, in particular, promotes hyperkeratosis of the pilosebaceous duct. *C. acne, Staphylococcus epidermidis *and *Staphylococcus aureus, *in addition, activate the innate immune system via Toll-like receptors (TLRs) 2 and 4 present on keratinocytes and monocytes enhancing release of inflammatory mediators such as IL-1, IL-6, IL-8, IL-12, TNF-α, and matrix metalloproteinases [9]. C-reactive protein (CRP), an acute-phase reactant synthesized in the liver in response to injury, infections, or inflammations, serves as a stable biomarker of systemic inflammation [10]. Malondialdehyde (MDA) is one of the by-products of lipid peroxidation and marker of oxidative damage [11]. As limited work has been done considering all the parameters (Hs-CRP, IL-6 and MDA) simultaneously, in our tertiary care center, the present study focused on evaluating the serum levels of these markers in patients with acne and assessing whether a correlation exists with disease severity.

## Materials and methods

The cross-sectional study was conducted in the Department of Dermatology in collaboration with Department of Biochemistry, VIMSAR Burla. The study period was from August 2023 to January 2024. After ethical approval from the institution (144-2022/I-F-O/137, dated July 27, 2023), the study was initiated. Written consent was obtained from the patients/guardians of study participants. Using 90% prevalence rate of acne vulgaris, a sample size of 150 was determined based on the formula written below [2].

Sample size calculation

Sample size (*n*) is calculated as follows:

$n = \frac{(Z_{1 - \alpha/2})^2 \cdot p \cdot q}{d^2},$ 

where *n* is the desired sample size, $Z_{1 - \alpha/2}$ the critical value, and a standard value for the corresponding level of confidence (at 95% confidence interval (CI) or 5% level of significance (type-I error), it was 1.96), *P* is prevalence based on earlier research, *q*=1 − *p*, and *d* is the margin of error or precision [12].

Inclusion criteria

All diagnosed cases of acne vulgaris between the age of 15 and 35 years of both sexes and those who consented to the study were included in the study.

Exclusion criteria

Patients suffering from other inflammatory conditions like psoriasis, acne fulminans, acne conglobate, rheumatic disorder, cancer, heart disease, diabetes mellitus, polycystic ovary syndrome, metabolic syndrome, hyperlipidemia, recent anti‑acne therapy, and receiving any kind of hormonal or antioxidant therapies, taking statins, fabric acid derivatives or niacin, and Vitamin D, in isotretinoin therapy (within one month), or pregnant, lactating mothers, indulging in a sedentary lifestyle, smoking, alcoholism and with auto-immune disorders were excluded.

Clinical assessment

Detailed history was obtained, and clinical examinations of the study participants visiting the Dermatology outpatient department (OPD) were performed by a dermatologist. The preliminary screening included occupational history and detailed family history of acne, sunscreen use, hormone therapy, occupation, and other relevant variables such as smoking, drinking alcohol, and food habits.

The Global Acne Grading System (GAGS) was used to evaluate the severity. Based on this, patients were categorized as mild (1-18), moderate (19-30), severe (31-38), and very severe (>39) [13]. As per GAGS criteria, the total body surface was divided into six areas: forehead, cheek, nose, chin, chest, and back. Each area was allocated based on the ratio between surface area and the distribution of pilosebaceous unit density.

Biochemical analysis

Under aseptic precautions, 7 mL of venous blood was collected from the antecubital vein of the study participants, and serum was separated after centrifugation at 2000 rpm and stored at -70℃ in a refrigerator until evaluation. Serum high-sensitive C-reactive protein (Hs-CRP) level measured by nephelometer (3563, TSI Incorporated, Shoreview, MN) and serum IL-6 and MDA by enzyme-linked immunosorbent assay (ELISA) (Erba, Dubai Science Park, Dubai).

Statistical analysis

Data were arranged systematically and analyzed using IBM SPSS Statistics, version 21.0 (IBM Corp, Armonk, NY). Quantitative data were expressed as a mean±standard deviation (SD). Qualitative data were shown as frequency and percentage. The effect sizes (0.089-4.15), 95% confidence interval and odds ratio (1.93, Cohen's d test) were noted. An unpaired t-test was applied to analyze between two variables, and analysis of variance (ANOVA) test was applied for analysis of more than two variables to assess the severity of the disease. The correlation of Hs-CRP, IL-6 and MDA with disease severity was analyzed using Pearson’s correlation coefficient. A p-value <0.05 was considered significant.

## Results

**Figure 1:**
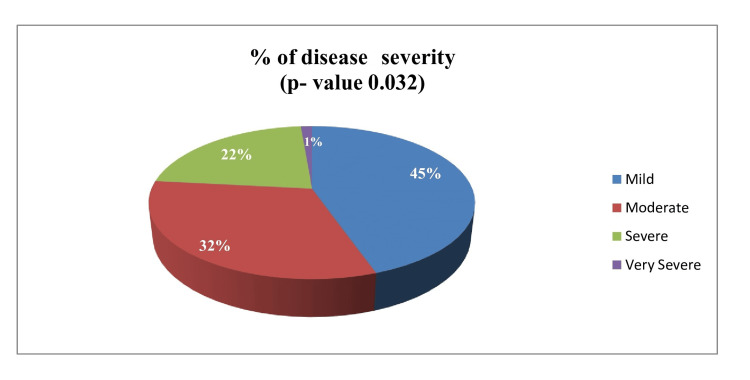
Percentage of disease severity

The demographic characteristics of patients with acne vulgaris are shown in Table 1. The study included 150 patients, of whom 81 were women (54%) and 69 were men (46%). The mean age of men and women were 20.59±1.3 and 22.25±0.4 years, respectively. The mean disease duration (in years) was 4.6±0.6. A positive family history of acne was noted in 66 (44%) women and 48 (32%) men. In this study, 98 (65.33%) study participants had a normal BMI. However, the number of overweight, obese, and underweight subjects were 35 (23.33%), 12 (8%) and 5 (3.33%), respectively. Furthermore, regarding the severity of acne vulgaris, 44.66% were mild, 32% were moderate, 22% were severe, and 1.33% were very severe, as shown in Figure 1. The most common lesion site was the face in 92 (61.33%) participants, followed by the chest (43, 28.66%) and back (15, 10%), as shown in Table 1. The mean age and BMI were not statistically significant. However, sex (p=0.009), disease severity (p=0.032), acne site (p=0.041), effect size (1.73, 95% confidence interval and power 80%), family history (p=0.002), occupation (p=0.032), and food habits (p=0.007) were statistically significant predictors (p<0.05).

**Table 1:** Sociodemographic parameters of study participants *N*=150 refers to the number of participants in the study; SD: standard deviation; BMI: body mass index; p-value<0.05, significant, NA: not applied.

Characteristic		Acne Patients (N=150)	p-value
Age in years (Mean±SD)	Men	20.59±1.3	0.21
	Women	22.25±0.4	
Gender, N (%)	Women	81 (54%)	0.009
	Men	69 (46%)	
Age group (years)	15-24	115 (76%)	0.061
	25-35	35 (23.33%)	
BMI (kg/m^2^) (WHO Indian standard)	Normal or less (18.5 ≤ 24.9)	98 (65.33%)	0.073
	Overweight (≥25-29.9)	35 (23.33%)	
	Obese (≥30)	12 (8%)	
	Underweight (≤8.5)	5 (3.33%)	
Family history of acne, N (%)	Women	66 (44%)	0.002
	Men	48 (32%)	
Site of acne, N (%)	Face	92 (61.33%)	0.041
	Chest	43 (28.66%)	
	Back	15 (10%)	
Disease severity, N (%)	Mild	67 (44.66%)	0.032
	Moderate	48 (32%)	
	Severe	33 (22%)	
	Very severe	2 (1.33%)	
Occupation	Student	71 (47.33%)	0.032
	Laborer	42(28%)	
	Working in office	28 (18.66%)	
	Others	9 (6%)	
History of taking junk foods N (%)	77 (51.33%)		0.007
Duration of disease, Year (mean ±SD)	4.6±0.6		NA

A comparison of inflammatory markers and oxidative stress with sex distribution is shown in Table 2. The mean Hs-CRP (2.40±0.52 mg/L) was slightly higher in men than in women (p=0.046, effect size 0.54, confidence interval 95% and power 80%). Similarly, higher IL-6 (6.21±0.47 pg/ml) was observed in men. The level of MDA (3.57±1.31 nmol/ml) was higher in men (p value-0.042, effect size 0.62, confidence interval 95% and power 80%) than women.

**Table 2:** Comparison of inflammatory markers and oxidative stress marker with sex distribution n=number of patients, Hs-CRP=high-sensitive C-reactive protein, IL-6=Interleukin 6, MDA=malondialdehyde

Parameter	Acne patients (men, n=69)	Acne patients (women, n=81)	p value
Hs-CRP (mg/l)	2.40±0.52	2.1±0.6	0.046
IL-6 (pg/ml)	6.21±0.47	6.12±0.39	0.512
MDA (nmol/ml)	3.57±1.31	2.71±1.42	0.042

Inflammatory markers (Hs-CRP and IL-6) and oxidative stress markers (MDA) in comparison to different degrees of acne are depicted in Table 3. A significant result with a p-value of 0.001 was obtained. The serum level of the inflammatory marker Hs-CRP level was 1.65± 0.92 mg/L in mild, 2.64±0.80 mg/l in moderate and 3.0±0.10 mg/L in severe groups. The IL-6 level was 3.02±0.93 pg/ml, 6.94±1.70 pg/ml and 8.75±1.63 pg/ml in mild, moderate and severe groups, respectively. The oxidative marker, MDA level, was also higher in the severe group (5.31±1.89 nmol/ml) than mild (1.33±1.05 nmol/ml) and moderate (4.07±1.20 nmol/ml) groups.

**Table 3:** Comparison of Inflammatory markers and oxidative markers with disease severity n= number of patients, Hs-CRP: high-sensitive C-reactive protein, IL-6: interleukin 6, MDA: malondialdehyde

Parameters	Mild (n=67)	Moderate (n=48)	Severe (n=35)	p value
Hs -CRP (mg/l)	1.65± 0.92	2.64±0.80	3.0±0.10	0.001
IL-6 (pg/ml)	3.02±0.93	6.94±1.70	8.75±1.63	
MDA (nmol/ml)	1.33±1.05	4.07±1.20	5.31±1.89	

The correlation between inflammatory markers, oxidative stress markers, and different degrees of acne vulgaris is shown in Table 4. The ‘r’ values of mild, moderate, and severe were positively correlated with r values of 0.001, 0.044, and 0.021, respectively, in the case of Hs-CRP. Similar results were observed for IL-6 and MDA levels. However, Hs-CRP (r=0.137, p=0.009), IL-6 (r=0.719, p=0.008), and MDA (r=0.316, p=0.007) levels were significantly correlated in the severe group compared to the mild and moderate groups.

**Table 4:** Correlation of Inflammatory and oxidative stress markers with different degree of Acne vulgaris Hs-CRP: high-sensitive C-reactive protein; IL-6: Interleukin 6; MDA: malondialdehyde, r: Pearson coefficient.

Parameters	Mild		Moderate		Severe	
	r value	p-value	r value	p-value	r value	p-value
Hs-CRP (mg/l)	0.001	0.152	0.044	0.03	0.137	0.009
IL-6 (pg/ml)	0.044	0.121	0.184	0.005	0.719	0.008
MDA (nmol/ml)	0.021	0.051	0.102	0.009	0.316	0.007

## Discussion

The prevalence of acne vulgaris varies by age, nation, and ethnicity, affecting approximately 9.4% of the global population, with the highest prevalence observed in adolescence [14]. Acne vulgaris is a chronic inflammatory skin condition involving the pilosebaceous unit, primarily due to colonization by *C. acnes*. This bacterium stimulates the release of pro-inflammatory mediators, such as chemokines, lipases, and proteases, leading to the recruitment of lymphocytes, monocytes, and neutrophils to the affected areas [15].

Despite extensive research, the exact etiopathogenesis of acne remains unclear [16]. However, oxidative stress has been identified as a key factor in the pathogenesis of acne vulgaris [17].

In the present study, the majority of patients with acne were within the age range of 15-24 years and had a female predominance (54%) with a positive family history in 44% of the cases. These findings are consistent with those of Puspita et al. (2021), who observed a higher prevalence of acne among females [18].

The elevated levels of Hs-CRP observed in severe-grade acne in our study may be attributed to the increased number of inflammatory lesions and the concurrent elevation of cytokines such as IL-6 and TNF-α, which stimulate the hepatic production of CRP [1]. CRP is a pentameric acute-phase protein that binds to phosphorylcholine in a calcium-dependent manner. During systemic inflammation, the levels rise significantly. This process is largely mediated by IL-6, primarily secreted by macrophages and adipocytes, which promotes hepatic CRP synthesis [19]. The advantage of taking highly sensitive methods to measure CRP has made it possible to detect a very small increase of this inflammatory marker.

Our data showed higher Hs-CRP levels in men. It may be potentially linked to lifestyle factors such as occupation, bizarre food habits, obesity, and sunlight exposure in males [20]. We also found that Hs-CRP was significantly elevated in moderate (2.64±0.80) and severe (3.0±0.10) acne, with a significant positive correlation, especially in the severe group (r=0.137, p=0.009), as measured by GAGS. These findings are consistent with those of Nauli et al. (2023), who reported a positive correlation between CRP and acne severity [21].

IL-6 belongs to the gp130 family of cytokines and acts as a potent proinflammatory mediator, promoting hyperkeratosis of the pilosebaceous duct [22]. In the present study, IL-6 levels were significantly higher in patients with severe and moderate acne grades. This can be attributed to neutrophil degranulation at the site of inflammation, which releases proteolytic enzymes and reactive oxygen species (ROS) that form inflammatory lesions [8]. The abundance of neutrophils in pustular and cystic lesions likely explains the elevated IL-6 levels in patients with severe acne [23].

Our results showed a strong positive correlation between IL-6 and disease severity (r=0.719, p=0.008), which aligns with the findings of Stańkowska et al. (2020), who observed that IL-6 levels increased with acne severity. This is likely due to enhanced neutrophil recruitment and Th17-mediated inflammation, where Th17 cells are believed to be the major source of IL-6 in acne lesions [24].

Oxidative modification of proteins, lipids, and nucleic acids plays a significant role in the pathophysiology of various diseases [25]. Oxidized proteins can accumulate in tissues and contribute to various pathological conditions, including aging and neurodegenerative diseases. The measurement of these modified proteins in serum provides valuable insights into the overall oxidative status of an individual. Furthermore, the detection and quantification of specific oxidative modifications on serum proteins can help in the early diagnosis and monitoring of oxidative stress-related disorders.

MDA is a well-established marker of oxidative damage. Several studies have reported elevated MDA levels in patients with acne vulgaris [26]. The production of reactive oxygen species (ROS), particularly from neutrophils, accelerates lipid peroxidation, thereby damaging the follicular epithelium and promoting acne inflammation [27].

During lipid peroxidation, free radicals attack polyunsaturated fatty acids (PUFA) in cell membranes and plasma lipoproteins, forming lipid hydroperoxides (LOOH). LOOH is unstable and decomposes into secondary products, including 4-hydroxynonenal (4-HNE), hexanal, propanol, and MDA. But among these, MDA is the most stable and specific marker of lipid peroxidation [28].

The present study depicted a higher MDA level in men compared to women, consistent with earlier reports [29]. This may be due to gender-related differences in lifestyle factors such as occupation, bizarre food habits, obesity, sunlight exposure, and air pollution exposure in men influencing oxidative damage [25,26]. Additionally, MDA levels were markedly elevated in severe acne (5.31 ± 1.89 nmol/ml) compared to moderate (4.07±1.20 nmol/ml) and mild (1.33±1.05 nmol/ml) acne. We also observed a strong positive correlation between MDA levels and acne severity. These findings support the study by Sutono (2013), who reported the highest plasma MDA levels in patients with severe acne vulgaris [30].

Limitations

Despite the strengths of this study of using standardized GAGS scoring, some limitations exist. The study is cross-sectional in nature, which may restricts causal inference. Longitudinal studies are needed to establish temporal relationships between biomarker levels and acne progression. So, further studies with a larger population are warranted to validate these biomarkers as routine clinical tools and to explore their potential role in guiding treatment decisions in acne vulgaris.

## Conclusions

The findings of our study highlight a strong association between acne severity and elevated levels of inflammatory (Hs-CRP, IL-6) and oxidative stress (MDA) markers, supporting their role in the pathogenesis of the disease. Monitoring these reliable biomarkers early in the disease process could aid clinicians in assessing disease progression, identifying high-risk patients and tailoring more personalized and targeted therapeutic strategies.
